# Designing Personality-Adaptive Conversational Agents for Mental Health Care

**DOI:** 10.1007/s10796-022-10254-9

**Published:** 2022-03-02

**Authors:** Rangina Ahmad, Dominik Siemon, Ulrich Gnewuch, Susanne Robra-Bissantz

**Affiliations:** 1 Chair of Information Management, Institute of Business Information Systems, Technische Universität Braunschweig, Mühlenpfordtstraße 23, 38106 Braunschweig, Germany; 2 Department of Software Engineering, School of Engineering Science, LUT University, Mukkulankatu 19, 15210 Lahti, Finland; 3 Institute of Information Systems and Marketing, Karlsruhe Institute of Technology (KIT), Kaiserstraße 89-93, 76133 Karlsruhe, Germany

**Keywords:** Personality-adaptive conversational agent, Chatbot, Virtual assistant, Mental health, Artificial intelligence, Design science research

## Abstract

Millions of people experience mental health issues each year, increasing the necessity for health-related services. One emerging technology with the potential to help address the resulting shortage in health care providers and other barriers to treatment access are conversational agents (CAs). CAs are software-based systems designed to interact with humans through natural language. However, CAs do not live up to their full potential yet because they are unable to capture dynamic human behavior to an adequate extent to provide responses tailored to users’ personalities. To address this problem, we conducted a design science research (DSR) project to design personality-adaptive conversational agents (PACAs). Following an iterative and multi-step approach, we derive and formulate six design principles for PACAs for the domain of mental health care. The results of our evaluation with psychologists and psychiatrists suggest that PACAs can be a promising source of mental health support. With our design principles, we contribute to the body of design knowledge for CAs and provide guidance for practitioners who intend to design PACAs. Instantiating the principles may improve interaction with users who seek support for mental health issues.

## Introduction

The necessity for mental health-related services has increased rapidly (Luxton, 2020). The prevalence of mental health issues (such as anxiety, depression or loneliness) is increasing among people worldwide: One in four people in the world is affected by mental health issues at some point in their lives (WHO, 2017). Access to therapists is limited, resulting in an acute shortage of mental health workers globally (Ta et al., 2020; WHO, 2021). Waiting times to receive treatment, therefore, can profoundly affect a person’s quality of life (Luxton, 2014). Further barriers to treatment access include high costs and unequal access to health care resources, but also attitudinal barriers, such as stigma toward professional treatments and skepticism about the effectiveness of treatment (Wasil et al., 2021). Technological advancements, however, have improved access to mental health care services (Jones et al., 2014). Conversational agents (CAs) represent one emerging technology with the potential to help address the barriers that contribute to the unmet health care needs (Luxton, 2020). CAs are software-based systems designed to interact with humans through natural language (Feine et al., 2019). CA is the overarching and general term for software that interacts with users via written or spoken natural language, and includes systems such as chatbots, which provide text-based communication, and virtual assistants, which rely primarily on voice-based interaction (Diederich et al., 2022). Applying Artificial intelligence (AI)-based methods, such as machine learning and natural language processing (NLP), CAs have rapidly become more efficient and enable broad and often cross-topic conversations (Diederich et al., 2022; Nißen et al., 2021). A benefit of CAs is that they are accessible anywhere and available at any time to provide counseling and deliver therapeutic interventions, and they may therefore be a promising source of support helping people cope with mental health issues (Luxton, 2020; Ta et al., 2020). A recent review by Abd-Alrazaq et al. (2021) demonstrates that the usefulness level of mental health care CAs is perceived as high by patients and that they overall have positive perceptions of and opinions about the CAs. Though AI-based CAs are increasingly capable of handling highly complex tasks, such as social support and therapeutic decision-making in a human-like way, currently available CAs, however, do not live up to their full potential yet (Fiske et al., 2019; Graham et al., 2019). Capturing dynamic human behavior to an adequate extent, and providing responses and reactions tailored to users’ individual contexts, special interaction dynamics and personalities, still pose a challenge in designing CAs (Grudin & Jacques, 2019; McTear et al., 2016). The field of tailored health communication has long established the need to personalize conversation (Abd-Alrazaq et al., 2021; Smith et al., 2015), since language is a primary tool to understand patients’ experiences and express therapeutic interventions (Laranjo et al., 2018). Psychotherapy, in particular, is highly patient-centered in clinical practice, requiring skills such as observing patient behavior and adapting to their individual personalities and needs accordingly (Graham et al., 2019; Laranjo et al., 2018). In their interaction with individuals, contemporary CAs lack the ability to dynamically change their own personality to adapt to the user’s personality. Provided a CA has features normally associated with humans, such as the use of natural language or human-like appearance (Nass & Moon, 2000), human beings tend to treat computer systems as social entities and ascribe different personality traits to them (Nass et al., 1994). Thus, as individuals’ interactions with computers have a fundamental social nature, users feel more appreciated and comfortable interacting with the machines when they perceive CAs as more human-like (Moon & Nass, 1996; Nass et al., 1993). One way to ensure that a CA is perceived as human-like, is to provide the CA with the ability to dynamically change its personality traits. Since personality preferences differ from person to person (McCrae & Costa Jr, 1997), the personalization of CA conversation is highly important and required (Abd-alrazaq et al., 2019). Based on an established personality model, and with advances in NLP, we propose the concept of a personality-adaptive conversational agent (PACA). PACAs are able to recognize and express personality by automatically inferring personality traits from users, giving them the ability to adapt to the changing needs and states of users when establishing a personalized interaction with them. As personality differences are manifested in language use, engagement with users can be further enhanced through tailored conversation styles (Kocaballi et al., 2020). While there is a large body of descriptive knowledge on design elements or cues that can be adapted, there is a lack of prescriptive knowledge on ways in which to actually design PACAs. Therefore, we pose the following research question (RQ):

- *RQ: How to design personality-adaptive conversational agents (PACAs) to improve interaction with users in mental health care?*

To answer our RQ, we conduct a research project following a design science research (DSR) approach (Hevner et al., 2004). Our design approach is particularly anchored in two existing kernel theories, namely the five factor model of personality (McCrae & John, 1992) and the ‘computers are social actors’ paradigm (Nass et al., 1994). On the basis of these theories, we believe that PACAs have the potential to improve interaction with users by personalizing their health care (Luxton, 2014). To the best of our knowledge, there is no study that rigorously derives requirements from both literature issues and user stories to develop design principles, and that further evaluates the preliminary design principles by means of expert interviews, in order to propose an expository instantiation that translates the abstract knowledge, captured in the design principles, into applicable knowledge. Our results indicate that practitioners (e.g., designers, developers, etc.) who instantiate our design principles to create a PACA, receive a promising level of guidance to improve interaction with users in mental health care.

The remainder of this paper is structured as follows: In the section on related work, we give a brief overview of the history and current developments of mental health care CAs. We also provide an overview of current research and highlight the critical aspects in the use of mental health care CAs. In section three, we illustrate the theoretical foundations of our DSR project. Section four presents our research methodology in more detail. Section five contains our derived and evaluated design principles for PACAs, as well as a demonstration of an expository instantiation. Finally, we discuss our results, current limitations, and contributions of our work, and close with a conclusion.

## Related Work

Efforts to develop software-based systems within the health care environment have a long history. In fact, ELIZA – widely considered the first CA in history – first appeared in 1966 in a psychotherapeutic context (Weizenbaum, 1966). Though only developed for demonstration and not commercial purposes, the simple computer program was able to mimic a Rogerian psychotherapist communicating with people in an empathic manner (Weizenbaum, 1966). The interaction resulted in people ascribing human characteristics to it, and some psychiatrists seeing ELIZA’s potential computer-based therapy as a “form of psychological treatment” (Kerr, 2003, p. 305). The underlying technology of ELIZA was rather simple: By searching the textual input of its conversation partner for relevant keywords, the machine produced appropriate responses according to rules and directions based on scripts hand-crafted by the programmers (Natale, 2018; Peters, 2013). PARRY, another early example of a prototype CA developed in 1979, was designed to simulate and behave like a person with paranoid schizophrenia (Shum et al., 2018). The developers’ intention was to find out if other psychiatrists could determine a real paranoid patient from their computer model (Shah et al., 2016). PARRY was a rule-based CA and worked in a similar way as ELIZA, though with better language understanding capabilities (Shum et al., 2018).

Current interest in CAs for mental health care is also nascent, as can be seen in the growing number of online services offered by health care providers (Bendig et al., 2019). More than one-fourth of 15,000 mobile health apps focus on mental health diagnosis or support, according to the World Health Organization (Abd-Alrazaq et al., 2021). Over the last few years, three particularly prominent therapeutic mental health CAs based on AI technologies have emerged: Woebot (Woebot Health, 2021), Wysa (Wysa, 2021) and Tess (X2, 2021). These CAs are publicly available mobile phone applications aimed at helping people to manage symptoms of anxiety and depression by providing counseling services. Woebot is a CA that is built to assess, monitor and respond to users dealing with mental health issues (Woebot Health, 2021). The CA has a responsive way to intervene with in-the-moment help and provide targeted therapies based on cognitive behavioral therapy (Woebot Health, 2021). The AI chatbot Wysa is also based on cognitive behavioral therapy, but employs several other methods, such as behavioral reinforcement and mindfulness, to help clients with depression (D’Alfonso, 2020; Wysa, 2021). According to its developers, Wysa provides 24/7 high-quality mental health support (Wysa, 2021). The mental health chatbot Tess pursues a similar approach by being available 24/7 and delivering conversations in the same way that “a coach or friend would” (X2, 2021). Preliminary studies of the efficacy of all three applications have shown a significant reduction in depression and anxiety levels in the group of participants using the CAs (D’Alfonso, 2020). Though the presented CAs were developed to have specific personalities, they are not able to dynamically change these or to infer user personality in order to be more adaptive towards the users’ needs. Therefore, all three CAs represent a rather “one-size-fits-all” solution, by not adequately adapting to the specificities of their users.

Ever since research in the field of human-machine interaction stressed the importance of avoiding “one-size-fits-all” interactions, the customization or personalization of CAs to individual users have become an important research topic (Abd-Alrazaq et al., 2021). Though this topic is still in its infancy (Kocaballi et al., 2019), there has been an increased interest in studies addressing the aspect of personality adaptivity in CAs. Studies from Yorita et al. (2019), Kampman et al. (2019) and Ahmad et al. (2020a, b) show that it is technically feasible to develop a CA that adapts its own personality traits to match the identified traits of the user. Yorita et al. (2019) developed their CA specifically with the purpose of providing emotional support for the user. While the studies by Yorita et al. (2019), Kampman et al. (2019) and Ahmad et al. (2020a, b) primarily focus on text-based human-machine interaction, the work of Völkel et al. presents approaches to adapt a voice assistant’s personality to the user, in order to improve interaction experience (Völkel et al., 2020, 2021). Further studies by Ranjbartabar et al. (2018) and Zalake (2020) particularly deal with CAs that, among other factors, aim to adapt to user personality to reduce study stress (Ranjbartabar et al., 2018) or to promote anxiety coping strategies among college students (Zalake, 2020). The research of Wibhowo and Sanjaya (2021) concentrate on CAs for use in clinical psychology and psychotherapy: The authors developed a CA as a warning system to prevent individuals with borderline personality disorder from committing suicide. The previous studies mainly employ empirical methods to describe behaviors in interaction with these systems and consequently generate descriptive knowledge about the use and effectiveness of personality-adaptivity CAs. In addition, these studies all have different focuses, so there is still a research gap in providing prescriptive knowledge about how to design a PACA to improve interactions with users in mental health care.

Due to technological advancements, AI-based CAs have become increasingly capable of handling highly complex tasks with human qualities such as a higher autonomy of decision-making (Brendel et al., 2021) or expressing human-like feelings (Porra et al., 2019). Consequently, the application of CAs can have diverse impacts on individuals – both positive and negative. The field of mental health care raises ethical considerations by its very nature (D’Alfonso, 2020). Voices have therefore emerged from the research field asking for a reassessment of the potential “dark sides” of AI and the ethical responsibilities of developers and designers (Brendel et al., 2021; Porra et al., 2019). Critics specifically stress the caveats of creating and perfecting human-like CAs with simulated feelings, without considering long-term consequences for human beings, such as deep, emotional attachments (Ahmad et al., 2021a). However, human beings treat computer systems as social entities and ascribe different personality traits to them (Nass et al., 1994); hence, they feel more appreciated and comfortable interacting with the machines when they perceive CAs as more human-like (Moon & Nass, 1996; Nass et al., 1993). To ensure an authentic interaction experience, CAs therefore should be imbued with some degree of human-like features (Gnewuch et al., 2017). While advanced CAs are able to simulate conversations employing therapeutic techniques, it is “not on the near horizon” (D’Alfonso, 2020, p. 113) for CAs to replicate human therapists. In fact, researchers agree that mental health care CAs should be used primarily as support systems, since the interaction experience and relationship that develops between a therapist and a patient is considered a significant factor in the outcome of psychological therapy (D’Alfonso, 2020) and cannot easily be substituted by a machine. The role of CAs in mental health care, rather, is to address individuals in need of treatment who are not receiving any treatment at all due to various barriers (Bendig et al., 2019; Stieger et al., 2018). In this way CAs could provide low-threshold access to mental health care but also bridge the waiting time before approval of psychotherapy (Bendig et al., 2019; Grünzig et al., 2018). Mental health care CAs have the potential to create their own form of interaction experience with users, provided the CA responses to users are tailored to their individual personalities. As a result, a personalized interaction would improve health outcomes among care seekers (Luxton, 2014). Given the widespread interest in mental health care CAs and the lack of prescriptive knowledge on how to design PACAs, it is important to address this research gap for researchers, designers, developers and clinicians.

## Theoretical Background

### Computers Are Social Actors

Extant research has shown that humans treat computers as social actors (Nass et al., 1994). The underlying reason, according to the ‘computers are social actors’ paradigm, is that humans automatically respond to social cues from computers (e.g., human-like avatars) in ways similar to how they would respond to social cues from another person (e.g., facial expressions, gestures) (Nass et al., 1994). This behavior can also be observed when users interact with CAs (Schuetzler et al., 2018). The fundamental reason is that CAs engage with users in a uniquely human activity, that is, having a conversation in natural language. According to Fogg (2002), interactive language use is one of the most salient social cues triggering social responses from users. In addition, CAs can display many other social cues, such as human-like avatars, names, vocalization or gestures (Feine et al., 2019). Research has shown that when users respond to these social cues from a CA, they perceive it as more human-like and feel more comfortable interacting with it (Moon & Nass, 1996; Nass et al., 1993). Studies have also shown that incorporating social cues can positively influence various CA-related outcomes, such as trust, enjoyment, and satisfaction (Kocaballi et al., 2019; Liu & Picard, 2005; Schuetzler et al., 2018). And since human beings treat computer systems as social entities and ascribe different personality traits to them (Nass et al., 1994), they perceive CAs as more human-like, particularly when the CAs’ expressions are attuned to the users’ state (Liu & Picard, 2005). For example, depending on the strength of a CA’s language, on the expressed confidence level, as well as on the interaction order, participants attribute an extraverted or introverted personality to the CA (Moon & Nass, 1996; Nass et al., 1995). Current CAs are designed to have a predefined personality (e.g., extraverted) and thus are not able to dynamically change their personality traits based on who they are interacting with. However, research has shown that users’ individual personality differences interact with the CA’s personality (Al-Natour et al., 2005; Gnewuch et al., 2020). As a result, different users may prefer different CA personalities, indicating that personality-adaptive CAs may be a sensible choice to meet user needs and preferences.

### The Five Factor Model of Personality

Personality is loosely defined as the construct that differentiates individuals from each other, but at the same time makes a human being’s behavior, thoughts and feelings (relatively) consistent (Allport, 1961). The dispositional approach considers trait as the key concept of the field of personality. In order to measure an individual’s personality, a widely used classification of personality – the five factor model or “Big Five” – has been applied in research (McCrae & John, 1992). Compared to other existing personality models, this multifactorial model was found to be stable across cultures and observers, and provides a taxonomy for the systematic evaluation of individuals (Goldberg, 1993; McCrae & John, 1992). The five fundamental traits that have been identified in this context are openness (to experience), neuroticism (also known as emotional range), conscientiousness, agreeableness, and extraversion (McCrae & John, 1992). The five factor approach to the taxonomy of traits is based on natural language, and more precisely lexical resources (Bouchet & Sansonnet, 2012). The lexical hypothesis states that most socially relevant and salient personality characteristics have become encoded in the natural language (Boyd & Pennebaker, 2017). Thus, language (verbal and non-verbal) has been found to be a fundamental dimension of personality (Boyd & Pennebaker, 2017). Human language reflects the psychological state and personality based on the frequency with which certain categories of words are used, as well as on the variations in word usage (Boyd & Pennebaker, 2017; Golbeck et al., 2011; Yarkoni, 2010). Psychologists have documented the existence of such cues by discovering correlations between a range of linguistic variables and personality traits across a wide range of linguistic levels (Mairesse et al., 2007). Language use has furthermore been scientifically proven to be unique, relatively reliable over time, and internally consistent, and as Boyd and Pennebaker (2017, p. 63) further state: “Language-based measures of personality can be useful for capturing/modeling lower-level personality processes that are more closely associated with important objective behavioral outcomes than traditional personality measures.” In addition to a speaker’s semantic content, utterances convey a great deal of information about the speaker, and the more extreme a person’s personality trait, the more consistently that trait will be a factor in their behavior (Mairesse & Walker, 2010). For example, extraverts have been found to have a higher rate of speech, to speak more, louder, and more repeatedly, with fewer hesitations and pauses, have higher verbal output, and use less formal language, whereas people who are highly agreeable show a lot of empathy, agree and compliment more, use longer words and many insight words, and make fewer personal attacks on their interlocutor (Mairesse & Walker, 2010). The combination of personality-specific words that people use in everyday life are internally consistent, vary considerably from person to person and is predictive of a wide range of behaviors (Boyd & Pennebaker, 2017; Pennebaker, 2011). Current personality mining services apply AI, specifically NLP technologies, to automatically infer personality traits from an individual’s speech or text (Ferrucci, 2012). NLP techniques particularly subserve to the interpretation of massive volumes of natural language elements by recognizing grammatical rules (e.g., syntax, context, usage patterns) of a word, sentence or document (Ferrucci, 2012).

## Methodology

To generate design knowledge about the design of PACAs, we follow the design science research (DSR) approach proposed by Hevner et al. (2004). The overall goal of DSR projects is to generate rigorously derived and relevant design knowledge. Design knowledge thereby covers all aspects of the relationship between the problem and the solution space (Venable, 2006; Vom Brocke et al., 2020), and aims to create prescriptive knowledge that contributes to both the theory and practice of solving real-world problems (Hevner, 2020). By providing prescriptive solution-oriented design knowledge, DSR therefore represents a complement to descriptive research that explains the nature of a phenomenon or problem. Gregor and Hevner (2013) define descriptive knowledge as omega-knowledge, which includes “descriptions of natural, artificial, and human-related phenomena” and is “composed of observations, classifications, measurements, and the cataloging of these descriptions into accessible forms” (p. A2). In contrast, DSR also creates prescriptive knowledge about artifacts that address the phenomenon or problem (Gregor et al., 2007), which Gregor and Hevner (2013) define as lambda-knowledge. DSR should not only generate original solutions to existing problems, but also demonstrate the beneficial application of such solutions to the problem and show their broad implications (Baskerville & Pries-Heje, 2010; Vom Brocke et al., 2020), thus generating both omega and lambda-knowledge (Hevner, 2020). Prescriptive design knowledge (lambda-knowledge) can be represented in different ways, such as design principles (DPs), design features, or instantiations (Hevner, 2020; Vom Brocke et al., 2020), whereas descriptive knowledge (omega-knowledge) describes observations, measurements or classifications of phenomena and sense-making, such as patterns or natural laws (Gregor & Hevner, 2013). Hence, DSR projects involve an interplay of synergies between descriptive and prescriptive knowledge, as well as the use of existing knowledge and the production of new knowledge (Hevner, 2020).

**Fig. 1:**
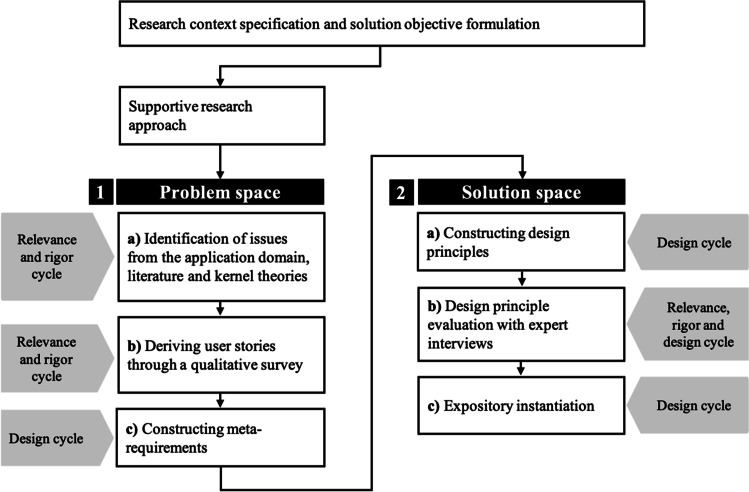
Research approach based on Iivari (2015), Möller et al. (2020) and Hevner (2007)

DPs as a form of formalized knowledge are gaining popularity in the field of information systems (IS) because they allow researchers to capture abstract or meta-knowledge that addresses a class of problems, rather than a single problem (Gregor et al., 2020; Iivari et al., 2021; Purao et al., 2020). To ensure practical relevance and applicability, DPs should imply accessibility, effectiveness, and, most importantly, guidance for action (Iivari et al., 2021). In our research, we follow an iterative and multi-step process that is fundamentally embedded in the three-cycle view of DSR (i.e., relevance cycle, rigor cycle and design cycle) (Hevner, 2007), to generate design knowledge for the design of PACAs for mental health care. The relevance cycle incorporates issues from the problem space in the study and places outcomes from the design cycle in evaluation and practice. The rigor cycle involves the use of kernel theories and scientific methods, as well as research experience and expertise, in the study, while also adding new knowledge created by the researchers to the growing knowledge base. The design cycle, as the core of DSR, includes construction activities for the development and evaluation of artifacts and design processes (Hevner, 2007). In our DSR project, we generate design knowledge in the form of DPs and an expository instantiation, by involving all three cycles multiple times. We follow the first strategy as proposed by Iivari (2015), in which we construct a meta-artefact (i.e., DPs) as a general solution concept, and then further adapt and follow the methodological components proposed by Möller et al. (2020) for the development of DPs in IS. These authors propose a supportive approach as well as a reflective approach that differ within “the point of artifact design and the logic of generating design principles” (p. 214). The two approaches of Möller et al. (2020) are similar to the two general strategies proposed by Iivari (2015) in that either generalizable design knowledge is created first, which is then concretized, or concrete design knowledge (e.g., design features or instantiations) is created first, which is then abstracted and generalized. However, the approach of Möller et al. (2020) focuses specifically on the construction of DPs and proposes a methodological procedure. We follow the supportive approach, in which DPs are “the provision of design knowledge in advance to support the design of an artifact before the design process takes place” (p. 214). Figure 1 shows our DSR approach based on Hevner (2007), Iivari (2015) and Möller et al. (2020).

Our DSR approach is divided into two main phases, addressing the problem space and the solution space (including the evaluation of the solution), which in turn consist of three sub-steps, respectively. To generate and evaluate a solution in the form of DPs for the identified problems, we use a mapping diagram. Mapping diagrams help to visualize the connection and derivation logic between DPs and meta-requirements, as well as between the problem space and the solution space (Möller et al., 2020). In this way, connections between and derivations of the individual aspects become clearer. The mapping diagram of the derivation and construction of the MRs and DPs can be found in the Appendix (see Fig. 4). An interplay of synergies between the rigor cycle, relevance cycle, and design cycle is reflected in each sub-step.

- Step 1: Problem Space

An important prerequisite for effective and practical design knowledge is a good understanding and description of the underlying problem space. To conceptualize the problem space, Maedche et al. (2019) propose considering and analyzing four key concepts of the problem space; stakeholders, needs, goals, and requirements. In our approach, the process of understanding and defining the problem space consists of three steps addressing the needs of the stakeholders and issues from the application domain, which we subsequently captured in requirements. To conceptualize the problem space, we followed three steps that build upon each other:

- Identifying issues from the application domain (relevance cycle), literature and kernel theories (rigor cycle)
- Deriving user stories (relevance cycle) through a qualitative survey (rigor cycle)
- Deriving meta-requirements (design cycle) through user stories, literature issues, application domain aspects, and kernel theories

**Fig. 2:**
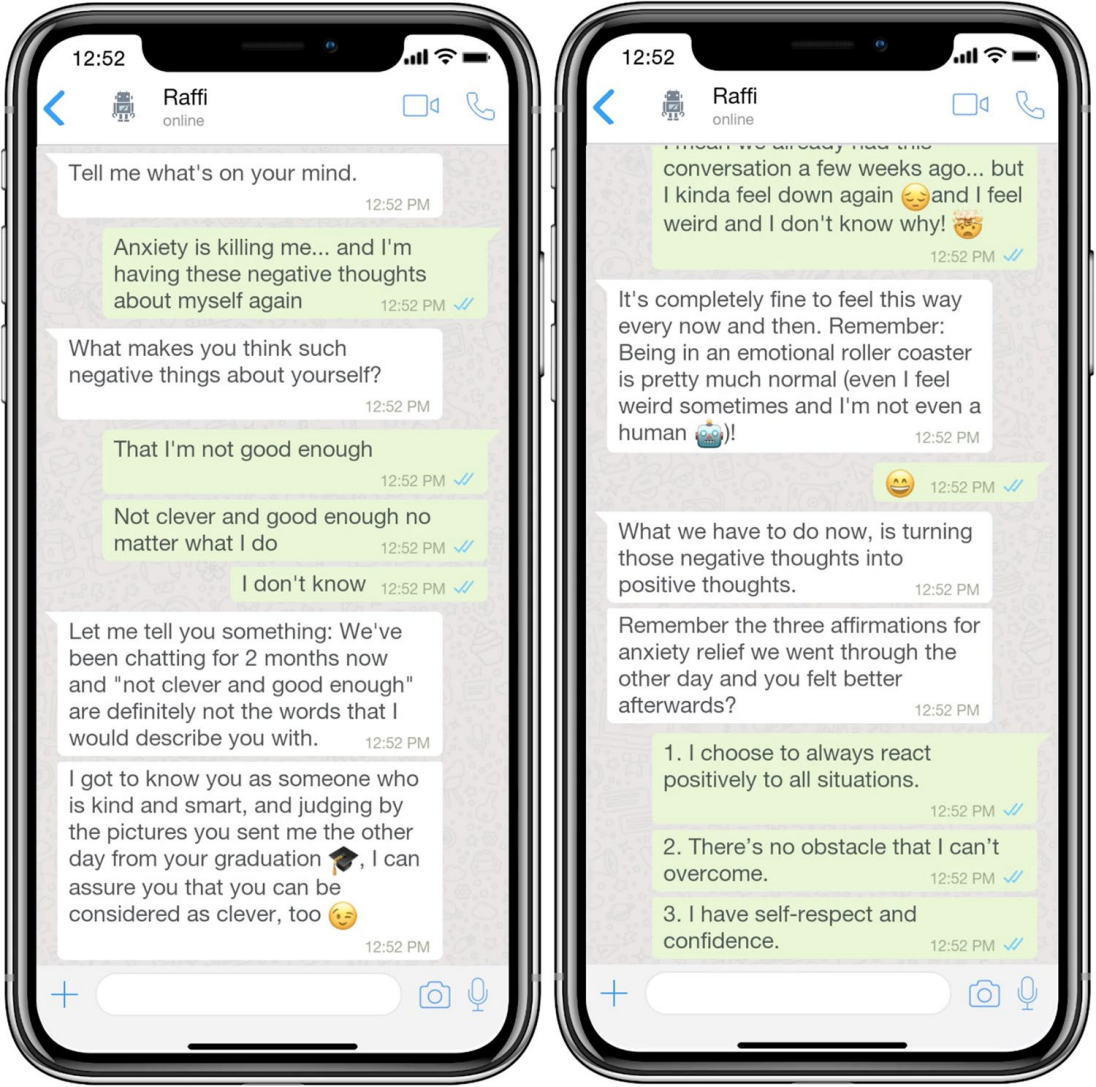
Mock-Up of the conversation between Raffi (PACA) and Jules (User)

We approached the problem space by reviewing extant literature and identifying current problems in mental health care. In a next step, and based on our kernel theories, we conducted a user survey to capture the perspective of potential users and to derive meta-requirements together with the literature issues. We followed an explorative approach and conducted a qualitative study (Babbie, 2020) using an open questionnaire with the aim to capture comprehensive user stories about PACAs in the context of mental health care. Our open questionnaire consisted first of an extensive explanation of the functionality and nature of a PACA to make sure participants from all backgrounds understood the concept. After that, we used an open questionnaire to capture user stories about the design of PACAs in the context of mental health care. These open questions asked for general requirements (e.g., mental health support, safety and privacy) and design-related requirements (e.g., behavior, communication style) (see Table 1 for an excerpt from the survey with sample responses). To help participants visualize what a conversation between a patient and a PACA might look like, we created a predefined chat record. For our simulated dialogue, we used the conversational design tool Botsociety, which allows prototyping and visualizing CAs. The conversation was provided in the form of a video. Figure 2 shows a mock-up of the simulated interaction. The video with the entire conversation can be viewed here: https://youtu.be/-sfSNJwCCI0

**Table 1:** Excerpt from the survey with sample responses

Question	Example response
Do you think the concept of a PACA - with the computer system having a personality and being able to adapt to the user’s personality - is useful/ helpful in mental health therapy?	*“Yeah I think it makes you feel less like you’re just sharing your emotions with an inanimate object, it makes you feel like there is some more meaning to sharing it with something that can at least pretend to care. It makes it feel more worthwhile to have it respond with something other than just a planned response.”*
Please comment briefly, why you think a specific trait is important in your opinion.	*“Being talkative will help in communicating feelings and affection is a trait that people with mental problems struggle to receive and comprehend.”*
In your opinion, in which of the roles should a mental healthcare PACA slip into? Please explain briefly why.	*“Looking at mental states means an emotional state of mind. A friend or companion are the closest to a patient and are therefore necessary.”*
What are, in your opinion, reasons that speak against communicating with a PACA? What concerns would you have in your interaction with the PACA?	*“I suppose it raises questions or suspicions about who could be reading or observing your conversations with the PACA. How secure and confidential is your information in the short and long term. It’s a question of trust.”*

The survey was distributed via our private network and the crowdsourcing platform Mechanical Turk (mTurk) and was carried out in December 2020. A total of 60 respondents participated in the study, producing more than 6865 words of qualitative data answering the open questions, which took roughly between 25 and 40 min to complete. Table 1 shows an excerpt from the survey with four open questions and example responses that were used for qualitative content analysis.

Participants (32 male, 28 female) were between 23 and 71 years old, with an average age of 36 years. In order to analyze the data, we followed a qualitative content analysis by coding the answers of the participants, which consisted mainly of inductive category forming (Mayring, 2014). The authors conducted the coding process independently, and whenever the results differed, we discussed the points of disagreement until we reached a consensus. In the last sub-step, we constructed meta-requirements from the captured user stories, the issues from the literature and the application domain.

- Step 2: Solution Space and Evaluation

Our proposed solution to the problem space identified in step 1 is a PACA for mental health care, for which we present design knowledge in the form of DPs. We constructed our DPs based on the meta-requirements, and then evaluated them with experts from the application domain. For the systematic derivation and evaluation of our solution space, we followed the following steps:

- Constructing design principles (design cycle).
- Evaluating design principles (design cycle) with expert (relevance cycle) interviews (rigor cycle).
- Designing an expository instantiation (design cycle).

Our DPs are formulated based on the approach proposed by Gregor et al. (2020, p. 2), who defined the anatomy of design principles so that DPs are “understandable and useful in real-world design contexts.” The authors point out the importance of including the concerned actors when formulating the DPs to complement the anatomy, with the aim that DPs should be “prescriptive statements that show how to do something to achieve a goal” (Gregor et al., 2020, p. 2). The anatomy of a DP consists of the aim, implementer, and user; context; mechanism; and rationale, and is presented in the form of heuristic rules. The evaluation of constructed artifacts such as DPs is an essential step in the design cycle to generate rigorous design knowledge. To evaluate our constructed DPs, we chose a qualitative approach in the form of expert interviews. To ensure relevance to the application domain, we selected experts, such as psychologists and psychiatrists, and had them evaluate the constructed DPs from a psychotherapist’s perspective. The criteria for selecting experts were a minimum of at least two years of professional experience in the care or therapy of mentally ill people, as well as a completed apprenticeship or studies in medicine, psychology, or another related field. The experts were selected and approached from our personal network. The work of EX1 (a psychologist) and EX6 (a social worker and therapist) particularly focus on youth care, while EX2 to EX5 are all psychiatrists who trained as psychotherapists and work with patients of all ages. EX2 deals specifically with geriatric psychiatry. The six experts were interviewed between March and April 2021. Each interview took between 50 and 80 min. Table 2 shows an overview of the interview panel, including the experts’ education and professional background.

**Table 2:** Expert panel for the DP evaluation

ID	Gender	Age	Education	Professional Background
EX1	Female	31	Studied psychology and has five years of working experience.	Works in the in-patient youth welfare in a therapeutic residential school with patients with mental disorders.
EX2	Male	38	Studied human medicine, specialized in neurology and psychiatry. Has nine years of working experience.	Works in a private clinic for psychiatry and psychotherapy (geriatric psychiatry) as a senior physician.
EX3	Female	29	Studied human medicine, completed an apprenticeship in psychotherapy. Has four years of experience.	Works as a medical specialist in psychiatry and psychotherapy in an outpatient and inpatient facility.
EX4	Male	28	Studied human medicine, completed an apprenticeship in psychiatry. Has two years of experience.	Works in a private clinic for psychiatry and psychotherapy.
EX5	Female	32	Studied human medicine, specialized in psychiatry. Has six years of experience.	Works in a private clinic for psychiatry and psychotherapy as a senior physician.
EX6	Female	35	Studied social work, has completed an apprenticeship as a therapist. Has eight years of working experience.	Works in residential youth care in a therapeutic residential home with patients with mental disorders.

The interviews were conducted with the support of a semi-structured interview guideline (Mayring, 2014). Interviews that follow a guideline are based on pre-defined questions that help orient the interviewer and thus ensure that all critical aspects of the scope of the interview are included. In addition, queries and deviations by the experts are possible (Mayring, 2014). The interview guide started with general questions about the profession of the experts, their professional experience, and their experience in dealing with CAs. This was followed by a detailed explanation of the concept of a PACA and follow-up questions by the interviewer to ensure that the concept was understood. Thereupon, all DPs were examined individually and evaluated by the experts, who were asked to recapitulate each DP in their own words, to again check whether they understood each DP. The experts were then asked to comment on each DP in terms of its relevance and to add possible missing aspects or highlight particularly relevant aspects. Subsequently, the audio recordings were transcribed and coded using MaxQDA (version 2020) qualitative data analysis software. The results were incorporated in the further development of the DPs. Despite the anatomy of a DP and different frameworks for the accumulation of design knowledge (Rothe et al., 2020), the reusability and use of DPs for practitioners is often challenging. Iivari et al. (2021) address this problem and specifically propose strengthening the actability and guidance for the implementation of DPs to maintain the practical relevance of DSR. Since DPs represent formalized design knowledge, we design an expository instantiation (Gregor et al., 2007) that translates the abstract knowledge captured in DPs into applicable knowledge. The next section presents the result of the two steps and their sub-steps.

## Designing PACAs for Mental Health Care

### Deriving Design Principles for PACAs

Within our problem space, we first identified several issues specific to our application domain (AD), as well as current literature issues (LI) related to CAs. The issues relating to the AD consequently establish the relevance of our research due to societal needs (i.e., unmet mental health care). The LIs reveal current issues and related theories of CAs regarding, for example, their interaction capabilities, which are captured in the literature. In contrast to the issues of the AD, the LIs originate from the knowledge base and show us, for example, why CAs have limited conversational ability. Both thus contribute to the relevance and rigor of our DSR project.

The World Health Organization reports that one in four people in the world is affected by mental health issues at some point in their lives (WHO, 2017). Particularly in times of humanitarian crisis, the necessity for health-related services increases rapidly (Luxton, 2020;Torous et al., 2020 ; WHO, 2021) **(AD1)**. As a result of this increase, there is a serious shortage of mental health workers globally (9 per 100,000 population), which in turn contributes to unmet health care needs (Luxton, 2020; Prakash & Das, 2020) **(AD2)**. Thus, there is a high need for offering IT-based mental health services to surpass the availability of healthcare workers and ease the burden on them. In addition to the high prevalence of mental health issues, there is a strong social stigma attached to mental illness (WHO, 2017). Therefore, patients with mental health issues are considered particularly vulnerable. Studies also show that the personality of a user plays a crucial role in the adoption of emerging technology that raises concerns about data security and privacy (Junglas et al., 2008). Since the field of mental health handles highly sensitive data, paying attention to the user’s personality is important. Hence, if patient safety is not addressed appropriately, a lack of privacy mechanisms, and thus a loss of trust, and could cause harm to people who exhibit sensitive mental health conditions (Luxton, 2020) **(AD3)**. Although CAs are considered an emerging technology with the potential to help address these issues, the adoption of CAs in mental health care has been slower than expected (Graham et al., 2020). Psychotherapy is a highly patient-centered clinical practice. This means that a successful conversation is particularly dependent on each patient’s individual dynamic behavior and the therapist’s ability to adapt to the patient’s specific personality in order to form a therapeutic relationship (Laranjo et al., 2018; Luxton, 2014). However, contemporary CAs do not capture user personality and individual dynamic human behavior to an adequate extent (Ahmad et al., 2021b; Yorita et al., 2019) **(LI1)**. As a result, the CA’s knowledge of the patient’s personality and behavior is restricted, and their ability to effectively adapt and consequently to develop rapport with the patient is limited. The needs and preferences of users while interacting can be fundamentally different. However, many CAs are focused on a “one size fits all”-approach instead of pursuing personalized communication. Contemporary CAs therefore insufficiently tailor responses to patients’ individual contexts, special interaction dynamics, and personality (Grudin & Jacques, 2019; McTear et al., 2016) **(LI2)**. This in turn results in decreased communication satisfaction, user engagement, and dialogue quality (Kocaballi et al., 2019). Another literature issue concerns current CAs’ limited ability to hold longer conversations or answer more complex questions (Chakrabarti & Luger, 2015; Clark et al., 2019). Although many CAs are increasingly capable of handling highly complex tasks with their natural language understanding capabilities, CAs are not sophisticated enough to recreate the richness of a conversation with a human therapist (Gaffney et al., 2019) **(LI3)**. As a consequence, CAs fail to sufficiently engage the patients and to respond with suitable advice (Kocaballi et al., 2019). A further identified literature issue is a CA’s degree of anthropomorphism: The perceived level of humanness can either have a positive or a negative impact on users (Prakash & Das, 2020) **(LI4)**. Hence, the degree of anthropomorphism of a CA can affect a patient’s judgments and attitudes (Kim et al., 2019), which can lead to the uncanny valley phenomenon - a feeling of uncanniness when CAs become too human-like (Kim et al., 2019). Table 3 sums up all ADs and LIs.

**Table 3:** Derived issues from application domain & literature

#	Application Domain (AD) and Literature Issues (LI)
AD1	Humanitarian crisis: Increased necessity for health-related services (Torous et al., 2020; WHO, 2021)
AD2	Shortage in health care providers and therapists (Luxton, 2020; 2020; WHO, 2021)
AD3	Patient safety: sensitive data and privacy concerns (Luxton, 2020, WHO, 2017)
LI1	Inability to adapt dynamically to a user’s personality (Ahmad et al., 2021b; Yorita et al., 2019)
LI2	No tailored responses to a user’s preferred communication style (Kocaballi et al., 2019)
LI3	Limited conversational ability (Chakrabarti & Luger, 2015; Clark et al., 2019)
LI4	Lacking adaptability to a user’s preferred degree of CA humanness (Kim et al., 2019)

Our underlying kernel theories – the five factor model and the ‘computers are social actors’ paradigm – are reflected in the user stories (US) derived from our qualitative study (see 1.b) of Fig. 1 and Table 1 for example responses). When the 60 participants recruited using mTurk and from our private network were asked to describe their ideal PACA for mental health care, they ascribed the virtual therapist with similar social cues and characteristics as they would have a human therapist. We aggregated the most common and repetitive USs into three categories: support, safety, and behavior. The category *support* sums up the ways in which the PACA could actively support the patient by improving its mental health service. The USs include around the clock availability **(US1)**, an easy conversation while interacting **(US2, US3)**, memorized conversations **(US4)**, a personalized interaction experience **(US5)**, competence **(US6)**, support and helpfulness for mental health therapy **(US7, US8)** and the ability to refer patients to human therapists **(US9)**. The category *safety* contains USs in which many participants stated that they want the PACA to be trustworthy **(US10)**, so they can build a relationship with the PACA **(US11)**. A basic requirement for this would be that the patient’s data should be secure and not misused by the PACA **(US12)**. The participants further indicated that there should be an option where the PACA-patient conversation is monitored by a human therapist **(US13)** so that the patient does not become dependent on the PACA and/or gets desocialized **(US14)**. A PACA should also be able to notice warning signals **(US15)**. The third category, *behavior*, refers to the characteristics that an ideal mental health care PACA should have, according to our participants. Although many of the participants agreed that a PACA should be and act rather human-like **(US16)**, as well as be able to communicate via voice and facial expressions **(US17)**, their preferences concerning the PACA’s communication style and personality traits varied strongly between participants. **US18-US28** in Table Table 4 sums up the aggravated PACA characteristics that were mentioned at least twice by various participants.

**Table 4:** Derived user stories from qualitative study

Category	#	User Story (US)
Support	US1	I want the PACA to be accessible and available for me 24/7.
	US2	I want the PACA to be easy to talk to like a friend/pen-pal/therapist.
	US3	I want the PACA to be able put me at ease.
	US4	I want the PACA to memorize our conversations.
	US5	I want a personalized interaction experience with the PACA.
	US6	I want the PACA to be competent.
	US7	I want the PACA to be supportive and motivate me.
	US8	I want the PACA to be helpful for my mental health therapy.
	US9	I want the PACA to be able to refer me to a human therapist.
Safety	US10	I want the PACA to be trustworthy.
	US11	I want to be able to build a relationship with the PACA.
	US12	I want my data to be secure and not misused.
	US13	I want the PACA to be able to be monitored by my therapist.
	US14	I don’t want to get desocialized and/or become dependent on the PACA.
	US15	I want the PACA to notice warning signals.
Behavior	US16	I want the PACA to be and act human-like.
	US17	I want the PACA to be able to have a voice and have facial expressions.
	US18	I (don’t) want the PACA to use emojis.
	US19	I want the PACA to have a rich vocabulary.
	US20	I (don’t) want the PACA to be formal.
	US21	I want the PACA to be calm, patient and receptive.
	US22	I want the PACA to be kind and polite.
	US23	I want the PACA to be humorous, witty and curious.
	US24	I want the PACA to be proactive and interactive.
	US25	I want the PACA to be confident and firm.
	US26	I want the PACA to be empathetic, sensitive and caring
	US27	I want the PACA to be open minded and attentive.
	US28	I want the PACA to be flexible and adaptive.

Based on our understanding of the problem space (i.e., AD, LI, US), we derived seven meta-requirements (MR) that ultimately led to six DPs for PACAs. After our expert evaluation, we adjusted the wording and refined the principles following the structure of Gregor et al. (2020). We elaborate on the MRs, as well as on the derived DPs, in the following section.

Mental health issues are increasing among people worldwide (AD1) and there is a dramatic shortage of mental health professionals (AD2), which is why users asked for an easily accessible and always available option (US1) to support them with their mental health issues (US3). To address this lack of human support for people in need, PACAs should always be available for their users, to put them at ease at any time (**MR1**). However, even when the PACA is available 24/7, not all users may actively seek help when they need it the most (AD3), because mental illness is still often deemphasized or stigmatized. Users also stated that they want a supportive and motivating PACA (US7) that supports them proactively (US24). To prevent users suffering in silence, PACAs should therefore take the initiative to reach out to their users on a regular basis (**MR2**). Based on these two MRs, we thus propose:

**DP1: Principle of Proactive Support**

For designers and developers to design personality-adaptive conversational agents (PACA) that provide mental health services to socially support patients independently of therapist, location and time, ensure that the PACA is accessible 24/7 and proactively checks in on the user on a regular basis, so they can receive support at any time.

Another MR we derived from the problem space is a PACA’s communication competence, particularly when it comes to developing and building a long-term relationship with a patient to improve well-being. Currently, CAs have limited conversational abilities (LI3), which is why users expressed their wishes that PACAs should be competent communication partners (US6) that can memorize past conversations (US4), have a rich vocabulary (US19), and are overall helpful for a patient’s mental health therapy (US8) by finding supportive and motivating words for the patient (US7) (**MR3**). We therefore propose:

**DP2: Principle of Competence**

For designers and developers to design personality-adaptive conversational agents (PACA) that provide mental health services to socially support users independently of therapist, location and time, provide the PACA with a domain-specific knowledge base and incorporate therapeutic techniques, so the user feels understood and perceives the PACA as competent.

The fourth MR specifically addresses patient safety (AD3). PACAs should protect patient data and always respect the users’ privacy, in ways similar to how human therapists build therapeutic relationships (US11) by being a trusted source of mental health support (US10) and by remaining strictly confidential (US12). Other safety requirements for a PACA are that it should support its users but not make them dependent on it (US14). Besides, a PACA should recognize critical trigger words (e.g., suicidal thoughts) and involve a human therapist (US9, US13). PACAs also need to analyze their patients’ data to learn more about their individual needs, preferences and personalities, to give tailored responses (LI2). In this regard, the PACA should be highly transparent towards its users (**MR4**). Hence, we propose:

**DP3: Principle of Transparency**

For designers and developers to design personality-adaptive conversational agents (PACA) that provide mental health services to socially support patients independently of therapist, location and time, ensure that the PACA is transparent in communicating patient safety and privacy issues, so that users trust the PACA with their health-related concerns and feel safe when sharing sensitive data.

One important aspect that users mentioned frequently and couched differently, is the PACA’s social role (US2). Different people in a patient’s life can provide social support, including therapists, friends, and family members. The responses in our study showed that different people preferred different types of social roles for the PACA (US2): While some participants indicated that their ideal PACA should, for example, take on the role of a witty and humorous friend (US23) who interacts in a more informal manner (US20), others described their PACA as a virtual therapist who should be more confident and firm (US25), and more formal in appearance (US20). Therefore, PACAs should be able to take on a social role (e.g., friend, therapist) based on the user’s individual needs and preferences (**MR5**). We consequently propose:

**DP4: Principle of Social Role**

For designers and developers to design personality-adaptive conversational agents (PACA) that provide mental health services to socially support users independently of therapist, location and time, allow users to choose between different social roles so the PACA can take on the user’s preferred social role and adapt to their needs.

Today, CAs can largely be customized only by user input. Customization is also mostly limited to simple and external aspects of a CA (LI4). However, the perceived level of humanness, which can be expressed through verbal and non-verbal language, can have either a positive or negative influence on users (LI4). A large number of our participants, though, stated that they wish to have a personalized interaction with their PACA (US5) and want their PACA to be and act human-like (US16). They further expressed their wish to interact with a PACA that can communicate non-verbally via voice and/or facial expressions (US17). Therefore, adapting the degree of adaptable anthropomorphism of a PACA, as well as further options of communication via voice and/or facial/body expressions, is required **(MR6)**. We therefore propose:

**DP5: Principle of Anthropomorphism**

For designers and developers to design personality-adaptive conversational agents (PACA) that provide mental health services to socially support users independently of therapist, location and time, allow the users to choose what type of PACA they want to interact with (chatbot, voice assistant, embodied conversational agent), so they can determine the PACA's degree of anthropomorphism based on individual needs.

Successful psychotherapy depends on the therapist’s ability to adapt to the patient’s specific personality to form a therapeutic relationship (LI1–2). However, current CAs follow a “one size fits all” approach instead of pursuing personalized communication. This is in line with our study participants’ requirements, that is, a personalized interaction experience with the PACA (US5). In addition, users may have vastly different preferences for communication with a PACA. This observation aligns with the responses from our user stories (US18-US28). For example, while some participants preferred texting with an extraverted and witty PACA (US23), others indicated they would rather trust an introverted PACA that communicates in a calm and soothing voice (US21). In summary, our participants stated that they not only wish a personalized interaction (US5) but also a flexible and adaptive PACA (US28) Therefore, the personality of a PACA should be aligned with the users’ preferences (**MR7**). We propose the following principle:

**DP6: Principle of Personality Adaptivity**

For designers and developers to design personality-adaptive conversational agents (PACA) that provide mental health services to socially support users independently of therapist, location and time, imbue the PACA with language cues specific to different personality dimensions, to enable the PACA to adapt to the user’s preferred communication style and increase interaction quality.

### Evaluating Design Principles for PACAs

*DP1: Principle of Proactive Support* was rated as an important principle by all experts; both constant availability and regular, proactive check-ups were named as highly useful features for a PACA. EX5 and EX6 pointed out that around-the-clock availability and more regular check-ins are advantages that PACAs might have over human mental health professionals. As to whether the PACA should always act in a proactive manner, the experts almost unanimously stated that the majority of patients would most probably appreciate such a function. Specifically, patients with certain mental health conditions, such as anxiety disorders or depressions, are often not able to actively seek help, which is why a regular check-in by the PACA would be helpful. However, EX4 also noted that for serious psychiatric illnesses, such as schizophrenia or paranoia, a proactive PACA might be too intrusive, giving the patient the feeling of being observed and monitored in a way which would be not beneficial for their mental health state. EX3 and EX5 further indicated that the patient, or even their treating therapist, should be able to select the intervals at which the PACA should become proactive, as too much or too little intervention can influence the quality of the support. We therefore slightly adjusted the description for DP1 and now mention that the PACA should check in on the user on a *mutually agreed* regular basis (see Table 5).

The experts considered *DP2: Principle of Competence* as equally important as DP1, stating that a PACA that is used in a mental health care context most definitely needs to have domain-specific knowledge and should be able to use therapeutic techniques in conversation with the patient. EX3, EX4 and EX5 remarked that the inclusion of ‘therapeutic techniques’ does not necessarily mean incorporating techniques that are specific to certain schools of psychotherapy (e.g., cognitive behavioral therapy, psychodynamic psychotherapy) in the PACA, but rather that the PACA should able to understand the patient’s individual concerns and then dynamically respond with suitable advice. However, four of the experts were slightly skeptical whether PACAs could be designed so that they are sophisticated enough to recreate the richness of a conversation with a human therapist. While EX2 and EX4 highlighted the importance of “reading between the lines” in order to react to warning signals, EX3 indicated that the PACA’s way of communication should also be perceived as “authentic” by showing understanding, otherwise patients could easily get disappointed by the PACA’s lack of perceived competence. The experts agreed with the formulation of DP2, and therefore we did not change the description.

All six experts considered *DP3: Principle of Transparency* a fundamental prerequisite for ensuring patient safety. According to EX1 and EX6, a PACA should be under a duty of professional secrecy equivalent to that of a healthcare professional. EX2 further stated that, in order to build trust with the patient, a PACA should be absolutely transparent about what happens with the patient’s data, because, as EX4 further remarked, a PACA would otherwise “risk losing their patients’ trust.” EX5 pointed out that, although it is crucial to inform the patient about the medical confidentiality at the beginning of the very first session, it does not necessarily have to be an integral part of every session. However, a PACA should always be able to address the privacy terms when asked by the user. EX4 suggested reminding the user at regular intervals that writing with the PACA is a “safe space.” Moreover, all experts highlighted the importance of building a steady therapeutic relationship, as rapport and trust can only be built over a longer period. To achieve this, a PACA must be considered a safe space for the users. The wording of DP3 was accepted by the experts, hence nothing was changed in the description.

The preliminary version of *DP4: Principle of Social Role* was not formulated clearly enough, since the majority of experts needed some explanation. It was not intuitively clear what we meant by *social role*, so we added specific examples (“e.g., friend, therapist etc.”) to the DP4 description. Once we mentioned the examples, the meaning was clear and the experts did not need further explanations. The principle was rated by the experts as valuable, however not as important as the previous DPs. EX3 and EX1 mentioned the importance of specific roles that people reflect for patients and approved the idea of having a PACA that reflects a specific role. EX4 and EX6 argued that, from a therapeutic perspective, it might not always be effective for the patients’ therapy progress if the PACA continuously takes on the role of a friend who never “counters or addresses unpleasant topics.” EX2 further indicated that specific roles or genders can be associated with fear or aggression. To avoid this, EX5 suggested that the PACA should be able to change its social role situationally, even within one session. EX2 further assumed that a patient who can choose between different social roles for their PACA would be more likely to use the service than if the option did not exist. After receiving this feedback, we elaborated on DP4 by adding that the user can also switch between certain social roles to promote therapy progress (see Table 5).

**Table 5:** Evaluated design principles for PACAs

#	Title	Design Principles (DPs)
DP1	Principle of Proactive Support	For designers and developers to design personality-adaptive conversational agents (PACA) that provide mental health services to socially support patients independently of therapist, location and time, ensure that the PACA is accessible 24/7 and proactively checks in on the user on a mutually agreed regular basis, so they can receive support at any time.
DP2	Principle of Competence	For designers and developers to design personality-adaptive conversational agents (PACA) that provide mental health services to socially support users independently of therapist, location and time, provide the PACA with a domain-specific knowledge base and incorporate therapeutic techniques, so the user feels understood and perceives the PACA as competent.
DP3	Principle of Transparency	For designers and developers to design personality-adaptive conversational agents (PACA) that provide mental health services to socially support patients independently of therapist, location and time, ensure that the PACA is transparent in communicating patient safety and privacy issues, so that users trust the PACA with their health-related concerns and feel safe when sharing sensitive data.
DP4	Principle of Social Role	For designers and developers to design personality-adaptive conversational agents (PACA) that provide mental health services to socially support users independently of therapist, location and time, allow users to choose between different social roles (e.g., friend, therapist etc.) so the PACA can dynamically take on the user’s preferred social role, but can also switch between social roles that promote the user’s therapy progress.
DP5	Principle of Anthropomorphism	For designers and developers to design personality-adaptive conversational agents (PACA) that provide mental health services to socially support users independently of therapist, location and time, allow the users to choose the type of PACA they want to interact with (chatbot, voice assistant, embodied conversational agent), so they can determine the PACA’s degree of anthropomorphism based on individual needs.
DP6	Principle of Personality Adaptivity	For designers and developers to design personality-adaptive conversational agents (PACA) that provide mental health services to socially support users independently of therapist, location and time, imbue the PACA with language cues specific to different personality dimensions to enable the PACA to adapt to the user’s preferred personality and increase interaction quality.

The experts rated *DP5: Principle of Anthropomorphism* as of similar importance as DP4, stating that it promotes better adaptability to the individual user. From a psychotherapeutic perspective, the principle can be specifically beneficial when patients “cannot talk about their issues, but rather prefer to write” (EX2), or when patients “have difficulty reading emotions from text, non-verbal language can help” (EX4). EX6 further stated that some patients need to feel a PACA’s social presence, for example in the form of an embodied CA, to open up and feel comfortable. EX3, however, doubted the efficacy of this, as she was rather critical towards CAs that are too anthropomorphized, stating that it could lead to negative dependencies. She therefore indicated that DP5 must be viewed and designed with caution. EX1 remarked that the PACA’s level of humanness might affect how patients perceive the PACA’s competence. EX5 suggested that familiar voices can be helpful in crisis mode and thus can be considered a useful feature for the PACA. As DP5 was comprehensible and the experts agreed on the wording of the description, nothing was modified.

Concerning *DP6: Principle of Personality Adaptivity*, all experts pointed out that capturing a patient’s dynamic behavior and individual personality during therapy is an essential step towards forming a trustful therapeutic relationship. EX2 and EX3 explained that building rapport with patients usually takes several hours of therapy until the patients are able to slowly open up. In this context, EX4, EX2 and EX1 highlighted the importance of language, and stressed that the style in which they communicate with their patients, specifically, plays a crucial role in the quality of their interaction. All six experts stated that, in the context of mental health care, the most valuable feature with which to imbue a PACA is the ability to capture patient personality, just as a human therapist would do. EX3 stated that, although therapists have their own unique personality and therapeutic techniques, they very much act (to some degree) like a PACA by adapting to their patients’ personalities. Therefore, DP6 was rated as particularly important. However, EX4 and EX5 remarked that, for therapeutic purposes, the PACA should also be able to change its personality and communication style to a “provocative style” (EX4) to “break the patient through their reserve from time to time” and to “not become a people pleaser” (EX5). EX4 added that a PACA should be able to dynamically change its personality, if the goal is to achieve therapeutic progress. To make DP6 more comprehensible, we changed “preferred communication style” to “personality” (see Table 5). Table 5 summarizes the revised and final six DPs.

In general, the experts agreed that the six DPs cover all important criteria for designing a PACA for mental health care. They saw potential in designing PACAs to address the issues from the ADs; however, they also strongly emphasized that experts in psychotherapy should be involved in the design of mental health care PACAs. They further indicated that a user’s experienced level of mental health issue(s) can play an important role in whether a PACA can be effective or not. The psychiatrists stated in particular that realistically, a PACA alone would not be able to help users with severe mental health issues, but could rather be a useful tool for both the patient and their treating therapist. Though they were familiar with the terms *chatbot* and *digital assistant*, the experts admitted that visualizing a PACA with therapist-like capabilities would only be possible if they could “experience [it] first-hand, to see how it really works” (EX2).

### Expository Instantiation

**Fig. 3:**
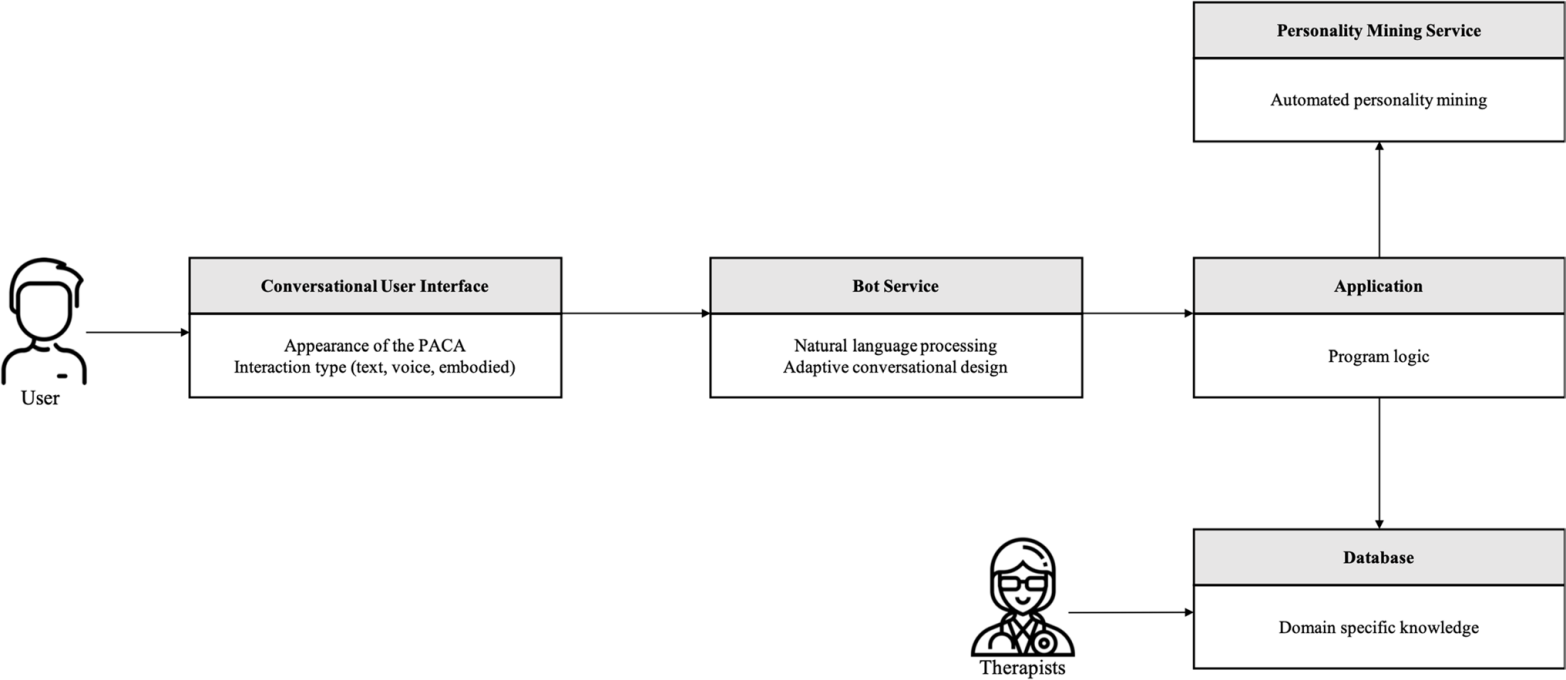
Expository instantiation

**Fig. 4:**
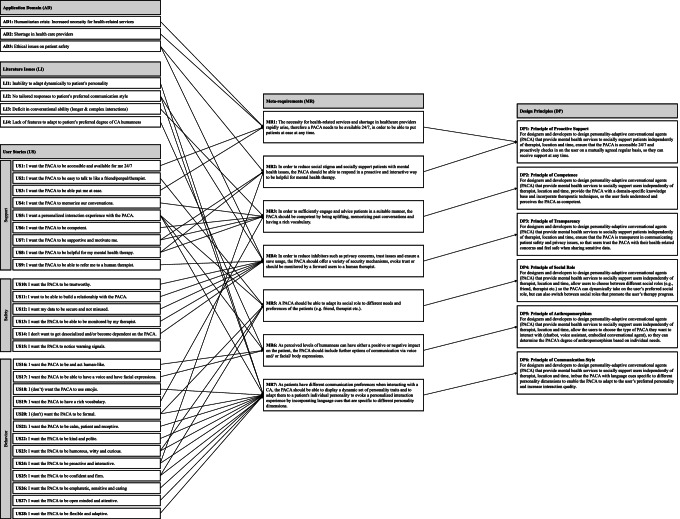
Mapping diagram of the derivation and construction of the MRs and DPs (Möller et al., 2020)

To show the applicability of our DPs and provide guidance for the implementation of a PACA, we developed an expository instantiation (Gregor et al., 2007; Iivari et al., 2021). Design knowledge, especially DPs, tends to be highly abstract and consequently cannot always be implemented easily and directly. Therefore, we transformed our defined DPs into an expository instantiation that can assist in “representing the design knowledge both as an expository device and for purposes of testing” (Gregor et al., 2007, p. 322). For this purpose, we modeled a PACA starting from the proposed DPs, so that system engineers and software developers have the abstract design knowledge of the DPs available in a transferable form. We opted for a graphical modeling for the specification, design, documentation and visualization of software components and interfaces to transfer our DPs in applicable form. In our expository instantiation, we decided to model the functionality of a PACA in a technology-independent representation. Since technologies change very quickly, especially in the field of AI (e.g., chatbot services), the model can serve as a blueprint for future solutions. Figure 3 shows the model diagram of a PACA for mental health support.

Interaction with the PACA takes place via a conversational user interface (CUI), which ensures continuous availability for the user and the possibility to proactively contact the user through push notifications, for example (DP1). Examples of typical CUIs include messaging applications, such as WhatsApp or Facebook Messenger, or a standalone service implemented on a website or in a mobile application. The CUI is also responsible for the representation of the PACA and its anthropomorphic features (DP5). To be able to display all cues desired by the user, the CUI should be able to provide both text and speech-based output and should be able to represent an embodied PACA (i.e., body language). Therefore, integration in a website or as a mobile application that allows a rich representation of a PACA is preferable. The bot service is responsible for the logical interaction with the user, and therefore is able to process natural language. In addition, the bot service can adapt its conversational design, which determines the style of communication and personality adaptivity (DP6). The conversational design is usually created by a conversational designer, who should co-develop it with a therapist in the case of a PACA. The conversational design is guided by the application logic, which in turn draws on the database and the results of the personality mining service. This functionality could be realized with services such as Google Dialogflow, IBM Watson Assistant, Amazon Lex, or Wit.AI.

The application is the core of the PACA and contains the general logic of the system. It accesses information from the knowledge base (database) and the information from the personality insight service to decide how to interact with the user. The application specifies not only the social role (DP4), but also the type of communication (DP6) and the degree of the anthropomorphism (DP5), which is the core of its adaptability. Together with the knowledge base and the identified personality of the user, it represents the competence of the PACA (DP2), which is reflected in the conversational design of the bot service and the CUI. The application should be implemented as platform-independently as possible and be able to communicate with the database, with the personality mining service, and with the bot service, which is why technologies or programming languages such as JavaScript, Python, Scala, Ruby, or Go are suitable. The personality mining service is responsible for analyzing the user’s messages and determining the personality traits of the user. It accesses the data that the user enters via the CUI, and analyzes it. The results are then passed on to the application. One approach is to analyze specific word usage and build statistical models that determine the personality traits of the user. The foundation of these procedures was laid by Pennebaker and Francis (1996) with the Linguistic Inquiry and Word Count (LIWC), which quickly gained prominence in linguistic analysis (Chung & Pennebaker, 2012; Pennebaker et al., 2015). Further improvements have been achieved using so-called word embeddings, such as Google’s word2vec or Stanford’s GloVe (Pennington et al., 2014). The advantage of such models is that semantic similarity between words is determined unsupervised, whereas LIWC relies on human judges and psychologists to determine the meaning of words (Arnoux et al., 2017; Rice & Zorn, 2021). Arnoux et al. (2017) suggest that models with word embeddings predict personality even better than models based on LIWC. IBM developed Watson Personality Insights, a commercial software package, as a service for ready-to-use personality predictions based on GloVe and Twitter posts (Arnoux et al., 2017). These models or services can be used to determine the personality traits of the user from their interactions. With the user’s personality traits identified, the PACA can adapt to the user according to the program logic (application).

The database contains the entire knowledge base of the PACA, ranging from communication forms, anthropomorphic expressions, and other cues to therapeutic methods and skills. The system also constantly learns from past interactions with all its users, as the application not only accesses the database, but also regularly updates it with information. The database also represents the learning aspect of a PACA, as it stores not only all therapeutic methods and knowledge bases, but also the knowledge gained from past interactions. For secure handling of the user’s sensitive data, it is important that all data is protected with the latest technology (DP3), such as multiple factor authentication and data encryption. This should not only be ensured across all components, but also be communicated to the user via the bot services, so that they can build trust and rapport with the PACA (DP3). We previously published a paper that describes the implementation of a PACA within another application domain (Ahmad et al., 2020a).

## Discussion

### Theoretical and Practical Contributions

We contribute to the body of design knowledge for CAs by providing evaluated DPs for the design of a PACA. The six derived DPs can be divided into two categories. The first category contains *DP1: Principle of Proactive Support, DP2: Principle of Competence* and *DP3: Principle of Transparency*. These three principles represent the foundation of a PACA and can be considered as basic prerequisites when designing a PACA. Proactive support, competence and transparency are particularly important design elements in the context of mental health care, as emphasized in our expert panel’s reports. However, it is the second category of DPs that transforms a CA into a PACA: *DP4: Principle of Social Role*, *DP5: Principle of Anthropomorphism* and *DP6: Principle of Personality Adaptivity* are the principles that enable adaptation in the form of customization and personalization to user preferences, and consequently provide a more tailored service based on users’ individual needs and personalities. As evaluated by the experts, DP6 in particular – which represents a necessary requirement for a PACA – is a crucial design element. The DPs do not build on each other and can be considered as stand-alone design elements. However, we believe that only a combination of all DPs provides the best possible results, since the six DPs cover all important criteria to design a PACA for mental health care.

Our work also offers several practical contributions. We generated design knowledge in the form of prescriptive knowledge, and we provide guidance for CA designers and developers. Based on our experts’ evaluation, we argue that instantiating our DPs to design a PACA should improve interaction with users who seek support for their mental health care. While it is unlikely that PACAs that mimic human interactions could ever completely replace human psychotherapists, they may be a promising source of support for a wide range of user groups and different situations. First, for users who want to receive social support as an everyday social interaction to reduce loneliness or prevent early mental health issues, PACAs can surpass availability of and ease the burden on health care providers by being accessible to provide counseling at any time. Especially in the current COVID-19 pandemic, the need for social support is of immense importance, and the lack of such support can otherwise profoundly affect a person’s quality of life. Second, for users who are undergoing therapy for more severe mental health issues and are receiving treatment from human professionals, a PACA may also be beneficial by delivering added value to therapeutic interventions. Since PACAs are not susceptible to forgetfulness or fatigue, they can be used as additional support systems for both the patient and the provider, by offering periodic check-ups, for example. In these cases, however, it is crucial for the PACA to be monitored by human professionals to ensure patient safety, as proposed by our experts.

### Limitations and Ethical Considerations

A number of limitations have to be considered with respect to our qualitative study and expert interviews. A number of our study participants indicated that they have no experience with mental health related therapies. Hence, the USs we gathered would probably have led to different requirements if all participants had a similar amount of experience with mental health related therapies. However, as our intention was to include a non-clinical population, that is people who might want to receive social support as an everyday social interaction, we did not exclude participants from our study who indicated that they have no experience with mental health therapies. Another limitation is the small number of experts who evaluated the DPs. All our experts have less than 10 years of experience in their field and are about the same age. Experts with significantly more years of experience (e.g., < 20 years) might have evaluated the DPs differently.

Despite the cost, reach, and scalability advantages of CAs over human counsellors and therapists, it is important to note that there may be several drawbacks to using CAs in mental health care. First, these CAs could cause users to isolate themselves even more from the outside world. Since it can be easier to establish a relationship with these CAs than with another human (e.g., a therapist), users may lose interest in meeting or spending time with with their human friends and family members (Skjuve et al., 2021). This is even more problematic as many CAs are deliberately designed to look and act like humans (e.g., by giving them names and avatars), which further blurs the line between humans and machines (Porra et al., 2019). Second, these CAs are developed and operated by companies with business interests. Therefore, some users worry that the sensitive information they present in their conversations with the CAs could be intentionally or unintentionally shared with third parties (Bae Brandtzæg et al., 2021). Finally, as users have no control over their own CA, they become very dependent on the company that operates the CA, and some are afraid that their CAs could be deleted at some point (Skjuve et al., 2021). When taking all these considerations together, it is important to keep these drawbacks in mind when designing PACAs. Nevertheless, we believe that the potential advantages outweigh the potential drawbacks when these CAs are designed appropriately.

## Conclusion

Though being a fruitful area with large practical potential, the adoption of CAs for mental health care is associated with some challenges. Literature issues involve the inability of current CAs to capture and adapt dynamically to user personality, as well as to provide responses and reactions tailored in accordance with users’ individual characteristics to an adequate extent. In addition, CAs do not live up to their full potential yet, as they have been shown to have limited conversational abilities, particularly when it comes to longer and more complex interactions. These issues, however, are important factors that need to be taken into consideration when addressing primary issues from the application domain, such as the increased necessity for health-related services, the shortage of health care providers, or the paramount importance of patient safety. Motivated by the lack of design knowledge for PACAs, we proposed and evaluated DPs for designing PACAs that improve interaction, specifically for users who seek support for their mental health. We focused on two steps to answer our research question: Based on our kernel theories we first identified current issues from the AD and LIs, as well as derived USs, through a qualitative study. Within this first step of our problem space, we then derived MRs. On the basis of step one, and as part of our solution space, we proposed DPs for PACAs in step two. We then conducted expert interviews with psychologists and psychiatrists to evaluate the derived DPs, and adjusted and refined our final DPs based on their feedback. In a last step, we transferred our defined DPs to an expository instantiation for the purpose of better visualization.

According to the DSR contribution framework proposed by Gregor and Hevner (2013), our work can be classified as an improvement, as we address a known problem with a new solution. We provide prescriptive design knowledge by deriving and evaluating DPs for PACAs in mental health care. Our DPs contribute to the body of design knowledge for CAs and provide guidance for practitioners, such as designers, developers, and mental health organizations, on how to design PACAs that can better support their users. Instantiating these principles may improve interaction with users who seek support for mental health issues. We believe that our design approach could also be a valuable starting point for the design of PACAs in other domains.

## Appendix

Figure 4
